# Schultz Index of Armchair Polyhex Nanotubes

**DOI:** 10.3390/ijms9102016

**Published:** 2008-10-29

**Authors:** Mehdi Eliasi, Nafiseh Salehi

**Affiliations:** Islamic Azad University Najafabad Branch, Isfahan, Iran; E-Mail: nsalehi@iaun.ac.ir

**Keywords:** Topological index, Wiener index, Schultz index, Armchair nanotube, Molecular graph, Distance, Carbon Nanotube

## Abstract

The study of topological indices – graph invariants that can be used for describing and predicting physicochemical or pharmacological properties of organic compounds – is currently one of the most active research fields in chemical graph theory. In this paper we study the Schultz index and find a relation with the Wiener index of the armchair polyhex nanotubes *TUV C_6_[2p; q]*. An exact expression for Schultz index of this molecule is also found.

## 1. Introduction

Topological indices are a convenient method of translating chemical constitution into numerical values that can be used for correlations with physical, chemical or biological properties. This method has been introduced by Harold Wiener as a descriptor for explaining the boiling points of paraffins [1–3]. If *d(u, v)* is the distance of the vertices *u*and *ν*of the undirected connected graph *G* (*i.e.*, the number of edges in the shortest path that connects *u* and *v*) and *V* (*G*) is the vertex set of *G*, then the Wiener index of *G* is the half sum of distances over all its vertex pairs (*u, v*):

$$W(G)=\frac{1}{2}\sum_{u\in V(G)}\sum_{\nu \in V(G)}d(u,\nu ).$$

A unified approach to the Wiener topological index and its various recent modifications is presented. Among these modifications particular attention is paid to the Hyper-Wiener, Harary, Szeged, Cluj and Schultz indices as well as their numerous variants and generalizations [4–10]. The Schultz index of the graph was introduced by Schultz [14] in 1989 and is defined as follows:

$$S(G)=\frac{1}{2}\sum_{u\in V(G)}\sum_{\nu \in V(G)}(\text{deg}(u)+\text{deg}(\nu ))d(u,\nu ),$$

where deg(*u*) is the degree of the vertex *u*.

The main chemical applications and mathematical properties of this index were established in a series of studies [12–15]. Also a comparative study of molecular descriptors showed that the Schultz index and Wiener index are mutually related [16–18].

**Figure 1.**
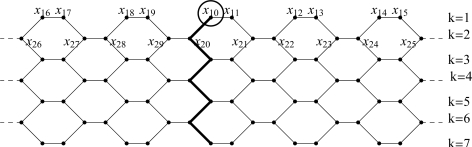
A *TUV C_6_[2p; q]* Lattice with *p* = 5 and *q* = 7.

Carbon nanotubes, the one-dimensional carbon allotropes, are intensively studied with respect to their promise to exhibit unique physical properties: mechanical, optical electronic etc. [19–21]. In [19], Diudea et al. obtained the Wiener index of *TUV C_6_*[2*p; q*], the armchair polyhex nanotube (see Figure 1). Here we find a relation between the Schultz index and Wiener index of this molecule. By using this relation we find an exact expression for the Schultz index of the same. The Appendix includes a Maple program [22] to produce the graph of *TUV C_6_*[2*p; q*], and to compute the Schultz index of the graph.

## 2. Schultz index of armchair polyhex nanotubes

**Figure 2.**
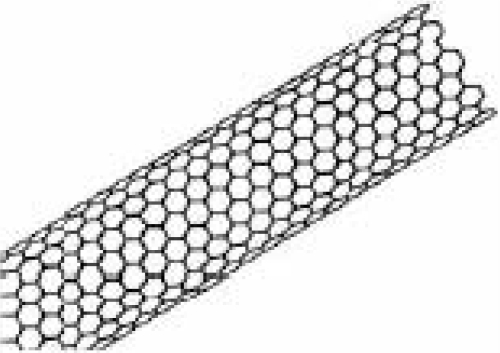
An armchair polyhex nanotube [19].

Throughout this paper *G* := *TUV C_6_*[2*p; q*]denotes an arbitrary armchair polyhex nanotube in terms of its circumference *2p* and their length *q*, see Figure 2. At first we consider an armchair lattice and choose a coordinate label for it, as illustrated in Figure 2. The distance of a vertex *u* of *G* is defined as

$$d(u)=\sum_{x\in V(G)}d(u,x),$$

the summation of distances between *v* and all vertices of *G*. By considering this notation the following lemma gives us a relation between the Schultz and Wiener index of *G*.

**Lemma 1**. For the graph *G* = *TUV C_6_*[2*p; q*]we have

$$S(G)=6W(G)-2\sum_{u\in level\;1}d(u).$$

**Proof:** For each *k* such that 1 ≤ *k* ≤ *q* put *A_k_* := {*u* ε *V (G)*│*u*; *level k*}( see Figure 2). Then

$$\begin{array}{l} S(G)\;=\frac{1}{2}\sum_{u\in V(G)}\sum_{\nu \in V(G)}\left(\text{deg}(u)+\text{deg}(\nu ))d(u,\nu \right)\\ \;\;\;\;\;\;\;\;\;\;\;\;=\frac{1}{2}\sum_{u\in V(G)}\sum_{\nu \in V(G)}\text{deg}(u)d(u,\nu )+\frac{1}{2}\sum_{u\in V(G)}\sum_{\nu \in V(G)}\text{deg}(\nu )d(u,\nu )\\ \;\;\;\;\;\;\;\;\;\;\;=\frac{1}{2}\sum_{u\in V(G)}\sum_{\nu \in V(G)}\text{deg}(u)d(u,\nu )+\frac{1}{2}\sum_{\nu \in V(G)}\sum_{u\in V(G)}\text{deg}(\nu )d(u,\nu )\\ \;\;\;\;\;\;\;\;\;\;\;=\frac{1}{2}\sum_{u\in V(G)}\text{deg}(u)\sum_{\nu \in V(G)}d(u,\nu )+\frac{1}{2}\sum_{\nu \in V(G)}\text{deg}(\nu )\sum_{u\in V(G)}d(u,\nu )\\ \;\;\;\;\;\;\;\;\;\;\;=\frac{1}{2}\sum_{u\in V(G)}\text{deg}(u)d(u)+\frac{1}{2}\sum_{\nu \in V(G)}\text{deg}(\nu )d(\nu )\\ \;\;\;\;\;\;\;\;\;\;=\sum_{u\in V(G)}\text{deg}(u)d(u) \end{array}$$

But

$$\text{deg}(u)=\{\begin{array}{lll} 2 & \text{if} & u\in A_{1}\cup A_{q}\\ 3 & \text{if} & otherwise. \end{array}$$

Also in the graph *G* it is clear that $\sum_{u\in A_{1}}d(u)=\sum_{u\in A_{q}}d(u)$. Therefore

$$\begin{array}{l} S(G)=\sum_{u\in V(G)}\text{deg}(u)d(u)=\sum_{u\in A_{1}\cup A_{q}}\text{deg}(u)d(u)+\sum_{u\in V(G)\backslash (A_{1}\cup A_{q})}deg(u)d(u)\\ \;\;\;\;\;\;\;\;\;\;\;=\sum_{u\in A_{1}\cup A_{q}}2d(u)+\sum_{u\in V(G)\backslash (A_{1}\cup A_{q})}3d(u)\\ \;\;\;\;\;\;\;\;\;\;=3\sum_{u\in V(G)}d(u)-2\sum_{u\in A_{1}}d(u)\\ \;\;\;\;\;\;\;\;\;=6W(G)-2\sum_{u\in A_{1}}d(u). \end{array}$$

This completes the proof.

To compute the *d(u)* in the graph *G*, when *u* is a vertex in level 1, we first prove the following lemma.

**Lemma 2.**The sum of distances of one vertex of level 1 to all vertices of level k is given by

$$\begin{array}{l} w_{k}:=\sum_{x\in level\;k}d(x_{10},x)=\sum_{x\in \;level\;k}d(x_{11},x)\\ \;\;\;\;\;\;\;\;\;\;\;\;\;\;\;\;\;\;\;\;\;\;\;\;\;\;\;\;\;\;\;\;\;\;\;\;\;\;\;\;\;\;\;\;\vdots \\ \;\;\;\;\;\;\;\;\;\;\;\;\;\;\;\;\;\;\;\;\;\;\;\;\;\;\;\;\;\;\;\;\;\;\;\;\;\;\;\;\;\;\;\;=\{\begin{array}{lll} 2p^{2}+k^{2}-2k-2p+1+H(p,k) & \text{if} & 1\leq k<p\\ p(p+2k-2) & \text{if} & k\geq p, \end{array} \end{array}$$

where

$$H(p,k)=\{\begin{array}{lll} 2p-1 & \text{if} & k+p\;\text{is}\;\text{even}\\ 2p & \text{if} & k+p\;\text{is odd}\text{.} \end{array}$$

**Proof:**We calculate the value of *w_k_*. We consider that the tube can be built up from two halves collapsing at the polygon line joining *x*_10_ to *x_q,_*_0_ (see Figure 2). The right part is the graph *G*_1_ which consists of vertical polygon lines 0, 1,. . . . . *p* and *x*_10_ is one of the vertices in the first row of the graph *G*_1_. The left part is the graph *G*_2_ which consists of vertical polygon lines (*p* + 1); (*p* + 2),. . . . , 2*p* –1. We change the indices of the vertices of *G*_2_ in the following way:

$$V(G_{2})=\{{\hat{x}}_{ji}|{\hat{x}}_{j,i}=x_{j,2p-i}\in V(G)\}$$

(See Figure 3)

We must consider two cases:**Case 1:** If *k ≥ p*. In the graphs *G*1 and for 0 *≤ *i* < *k** we have

$$d(x_{10},x_{k,i})=k+i-1.$$

Also in the graphs *G*_2_ and for 1 ≤.*i* < *k* we have

$$d(x_{10},{\hat{x}}_{k,i})=k+i-1.$$

So

$$\sum_{x\in \;level\;k}d(x_{10},x)=2\sum_{i=1}^{p-1}(k+i-1)+(0+k-1)+(p+k-1)=p(p+2k-2).$$

**Case 2:** If *k < p*. First suppose that 1 ≤ *i* < *k*. In the graphs *G*_1_ and *G*_2_ we have

$$d(x_{10},x_{k,i})=k+i-1=d(x_{10},{\hat{x}}_{k,i})=k+i-1.$$

Now suppose that *k* ≤ *i* ≤ *p*. Then in the graph *G*_1_ we can see that if *k* is odd, then

$$d(x_{10},x_{k,i})=\{\begin{array}{lll} 2i & \text{if} & i\;\text{is even}\\ 2i-1 & \text{if} & i\;\text{is odd } \end{array}$$

and if *k* is even, then

$$d(x_{10},x_{k,i})=\{\begin{array}{lll} 2i-1 & \text{if} & i\;\text{is even}\\ 2i & \text{if} & i\;\text{is odd}\text{. } \end{array}$$

Also in *G*_2_ we have

$$d(x_{10},{\hat{x}}_{k,i})=\{\begin{array}{lll} 2i & \text{if} & i\;\text{is even}\\ 2i+1 & \text{if} & i\;\text{is odd } \end{array}$$

if *k* is odd

$$d(x_{10},{\hat{x}}_{k,i})=\{\begin{array}{lll} 2i+1 & \text{if} & i\;\text{is even}\\ 2i & \text{if} & i\;\text{is odd } \end{array}$$

and if *k* is even.

All of this distances give us

$$\sum_{x\in \;level\;k}d(x_{10},x)=2p^{2}+k^{2}-2k-2p+1+H(p,k).$$

For other vertices we can convert those to *x*_10_ by changing transfer vertices and apply a similar argument by choosing suitable *G*_1_ and *G*_2_ and compute *w_k_*.

By a straightforward computation (if irem means the positive integer remainder) we can see:

$$\begin{array}{l} H(p,k)=2p-1+\text{irem(k}\;+\;\text{p},2)\\ \;\;\;\;\;\;\;\;\;\;\;\;\;\;\;\;\;=2p-1+\frac{1}{2}+\frac{1}{2}{\left(-1\right)}^{k-\text{irem}(\text{p},2)+1}, \end{array}$$

where

$$\text{irem}(\text{p},2)\;=\{\begin{array}{lll} 0 & \text{if} & p\;\text{is even}\\ 1 & \text{if} & p\;\text{is}\;\text{odd}\text{.} \end{array}$$

So, by Lemma 1, when 1 ≤ *k* ≤ *p*, we have

(1) $$w_{k}=2p^{2}+k^{2}-2k+\frac{1}{2}+\frac{1}{2}{\left(-1\right)}^{k-\text{irem}(\text{p},2)+1}.$$

Also in the graph *G*,

$$\begin{array}{l} d(x_{10})=\sum_{x\in level\;0}d(x_{10},x)+\sum_{x\in level\;1}d(x_{10},x)+\cdots +\sum_{x\in level\;q}d(x_{10},x)\\ \;\;\;\;\;\;\;\;\;\;\;\;\;\;=w_{1}+w_{2}+\cdots +w_{q}. \end{array}$$

So

$$d(x_{10})=d(x_{11})=\cdots =d(x_{2p-1,1})=w_{1}+w_{2}+\cdots +w_{q}.$$

This leads us to the following corollary:

**Corollary 1.** For each vertex *u* on level 1 we have

$$d(u)=w_{1}+w_{2}+\cdots +w_{q}.$$

Now suppose that *p > q*. Then by lemma 2 and equation (1) we have

$$\begin{array}{l} d(u)=\sum_{k=1}^{q}\left(2p^{2}+k^{2}-2k+\frac{1}{2}+\frac{1}{2}{\left(-1\right)}^{k-\text{irem}(\text{p},2)+1}\right)\\ \;\;\;\;\;\;\;\;\;\;\;=2p^{2}q+\frac{q^{3}}{3}-\frac{q^{2}}{2}-\frac{q}{3}+\frac{1}{4}{\left(-1\right)}^{-irem(p,2)+1+q}+\frac{1}{4}{\left(-1\right)}^{-irem(p,2)}. \end{array}$$

Also if *p ≤ q*, then by Lemma 1 and equation (1) we have

$$\begin{array}{l} d(u)=w_{1}+w_{2}+\cdots +w_{p-1}+w_{p}+w_{p+1}+\cdots +w_{q}\\ \;\;\;\;\;\;\;\;\;\;=\sum_{k=1}^{p-1}\left(2p^{2}+k^{2}-2k+\frac{1}{2}+\frac{1}{2}{\left(-1\right)}^{k-\text{irem}(\text{p},2)+1}\right)+\\ \;\;\;\;\;\;\;\;\;\;\;\;\;\;\sum_{k=p}^{q}p(p+2k-2)\\ \;\;\;\;\;\;\;\;=\frac{p^{3}}{3}+\frac{p^{2}}{2}-\frac{p}{3}-\frac{1}{4}{\left(-1\right)}^{-irem(p,2)+1+p}-\frac{1}{2}-\frac{1}{4}{\left(-1\right)}^{-irem(p,2)+1}+p^{2}q-pq+pq^{2} \end{array}$$

We summarize the above results in the following proposition

**Corollary 2.** For each vertex *u* on level 1, *d(u)* is given by

**Case 1:** p is even

$$d(u)=\{\begin{array}{lll} 2p^{2}q+\frac{q^{3}}{3}-\frac{q^{2}}{2}-\frac{q}{3}+\frac{1}{4}+\frac{1}{4}{\left(-1\right)}^{q+1} & \text{if} & p>q\\ \frac{p}{6}[2p^{2}+3p-2+6pq-6q+6q^{2}] & \text{if} & p\leq q \end{array}$$

**Case 2:** p is odd

$$d(u)=\{\begin{array}{lll} 2p^{2}q+\frac{q^{3}}{3}-\frac{q^{2}}{2}-\frac{q}{3}+\frac{1}{4}+\frac{1}{4}{\left(-1\right)}^{q} & \text{if} & p>q\\ \frac{p^{3}}{3}+\frac{p^{2}}{2}-\frac{p}{3}-\frac{1}{2}+p^{2}q-pq+pq^{2} & \text{if} & p\leq q \end{array}$$

**Theorem 1.** The Wiener index of *G* := *TUV C*_6_[*2p; q*] nanotubes is given by

**Case 1:** p is even

$$W(G)=\{\begin{array}{lll} \frac{p}{12}[3{\left(-1\right)}^{q+1}+3+24q^{2}p^{2}-8q^{2}+2q^{4}] & \text{if} & p>q\\ \frac{-{\text{p}}^{2}}{6}[8q-4p+p^{3}-4qp^{2}-4q^{3}-6q^{2}p] & \text{if} & p\leq q \end{array}$$

**Case 2:** p is odd

$$W(G)=\{\begin{array}{lll} \frac{p}{12}[3{\left(-1\right)}^{q}-3+24q^{2}p^{2}-8q^{2}+2q^{4}] & \text{if} & p>q\\ \frac{-p}{6}[-4p^{3}q-4pq^{3}-6q^{2}p^{2}+3+8qp-4p^{2}+p^{4}] & \text{if} & p\geq q \end{array}$$

**Proof:** See [19].

Now we are in the position to prove the main result of this section.

**Theorem 2.** The Schultz index of *G*:= *TUV C_6_[2p; q]* nanotubes is given by

**Case 1:** p is even

$$S(G)=\{\begin{array}{lll} \frac{p}{6}[-48q^{2}p+72p^{2}q^{2}+3{\left(-1\right)}^{q+1}+3-8q^{3}-12q^{2}+6q^{4}+8q] & \text{if} & p>q\\ \frac{-p^{2}}{3}[-18q^{2}p+3p^{3}-6p-12p^{2}q-12q^{3}+12q+4p^{2}-4+12pq+12q^{2}] & \text{if} & p\leq q \end{array}$$

**Case 2:** p is odd

$$S(G)=\{\begin{array}{lll} \frac{p}{6}[72q^{2}p^{2}+6q^{4}-12q^{2}-3+3{\left(-\right)}^{q}-48p^{2}q-8q^{3}+8q] & \text{if} & p>q\\ \frac{-p}{3}[-12p^{3}q-12pq^{3}-18p^{2}q^{2}+3+12pq-6p^{2}+3p^{4}+4p^{3}-4p+12p^{2}q+12pq^{2}] & \text{if} & p\leq q \end{array}$$

**Proof:** According to Lemma 1 we must calculate *6W(G)* –∑*_u_*_∈*level* 1_ *d(u)*. But by corollary 1 we have

$$d(u)=w_{1}+w_{2}+\cdots +w_{q}.$$

Since there are *2p* vertices on level 1 therefore

(2) S(G)=6W(G)-4pd(u)

Finally by replacing *d(u)* from corollary 1 in the equation (2) the result obtains.

## 3. Experimental Section

Tables 1 and 2 show the numerical data for the Schultz index in tubes *TUV C*_6_*[2p; q]* of various dimensions.

## Figures and Tables

**Figure 3.**
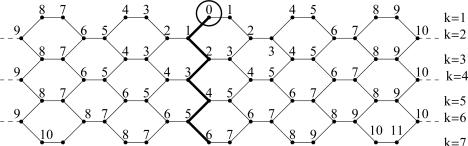
Distances from x01 to all vertices of *TUV C_6_[2p; q]* with *p* = 5 and *q* = 7.

**Table 1.** Schultz index of short tubes, *p* > *q*.

*p*	*q*	*S(G)*	*p*	*q*	*S(G)*
6	2	6912	5	2	4000
6	3	18366	5	3	10650
6	4	35424	5	4	20720
6	5	58656	9	5	193266
10	2	32000	9	6	288432
10	5	264160	9	7	404514
10	6	393440	9	8	542880
10	7	550560	15	8	2425440
10	8	736960	15	7	1823310
10	9	954400	15	6	1310160

**Table 2.** Schultz index of long tubes, *p* ≤ *q*.

p	q	S(G)	p	q	S(G)
4	4	10816	3	4	4752
4	5	18304	3	5	8262
4	6	28352	3	6	13104
4	7	41344	3	7	19494
4	8	57664	3	8	27648
10	21	6810400	11	11	1954502
10	22	7641600	11	12	2371952
10	23	8536800	11	13	2839524
10	24	9498400	11	14	3359312
10	25	10528800	11	15	3935030

## 4. Appendix

The following code is the MAPLE program [22] used to produce the graph of *TUHC**_6_**[2p; q]* and to compute the Schultz index of the graph.

> restart;with(networks):

> l:=proc(p,q) (*generating the graph *)

local G,i,j,k,ff,cc;G:=new();

  for i from 0 to (2*p–1) do

      for j from 1 to q do

      addvertex(a[i,j],G);

      end do;

  end do;

  for i from 0 to (2*p–1) do

        for j from 1 to (q–1) do

          addedge ({a[i,j],a[i,j+1]},G);

       end do;

  end do;

     for i from 0 to (2*p–2)/2 do

           for k from 1 to iquo(q,2) do

                addedge({a[2*i,2*k–1],a[2*i+1,2*k–1]},G);

           end do;

       end do;

  for i from 0 to (2*p–4)/2 do

    for k from 1 to iquo(q,2) do

      addedge({a[2&ast;i+1,2&ast;k],a[2&ast;i+2,2&ast;k]},G);

    end do;

  end do;

for ff from 1 to iquo(q,2) do

   addedge({a[2*p–1,2*ff],a[0,2*ff]},G);

  end do;

  if irem(q,2)=1 then

  for cc from 0 to 2*p/2–1 do

   addedge({a[2*cc,q],a[2*cc+1,q]},G); end do;

   end if ;return(G);

   end proc:

> m:=l(3,8):(#Graph G:=TUVC_6[2*3,8]#)

> t :=edges(m):

> ii:=vertices(m):

> T := allpairs(m,p):

> Sch:=proc(u)

   local b,o,pp;

   b:=0;

   for o in ii do

     for pp in ii do

         b:=b+ T[(pp,o)]*(vdegree(o,m)+vdegree(pp,m));

      end do;

   end do;

return(b/2);

   end proc:

> Sch(u); 27648(#The Schultz index of the graph #)
